# Supplementary material for: Differences in Compositions of Oral and Fecal Microbiota between Patients with Obesity and Controls
Source: Medicina (Kaunas). 2021 Jun 30;57(7):678. doi: 10.3390/medicina57070678 (PMC8306358; doi:10.3390/medicina57070678)

Figure S1. Alpha diversity for oral microbiota.

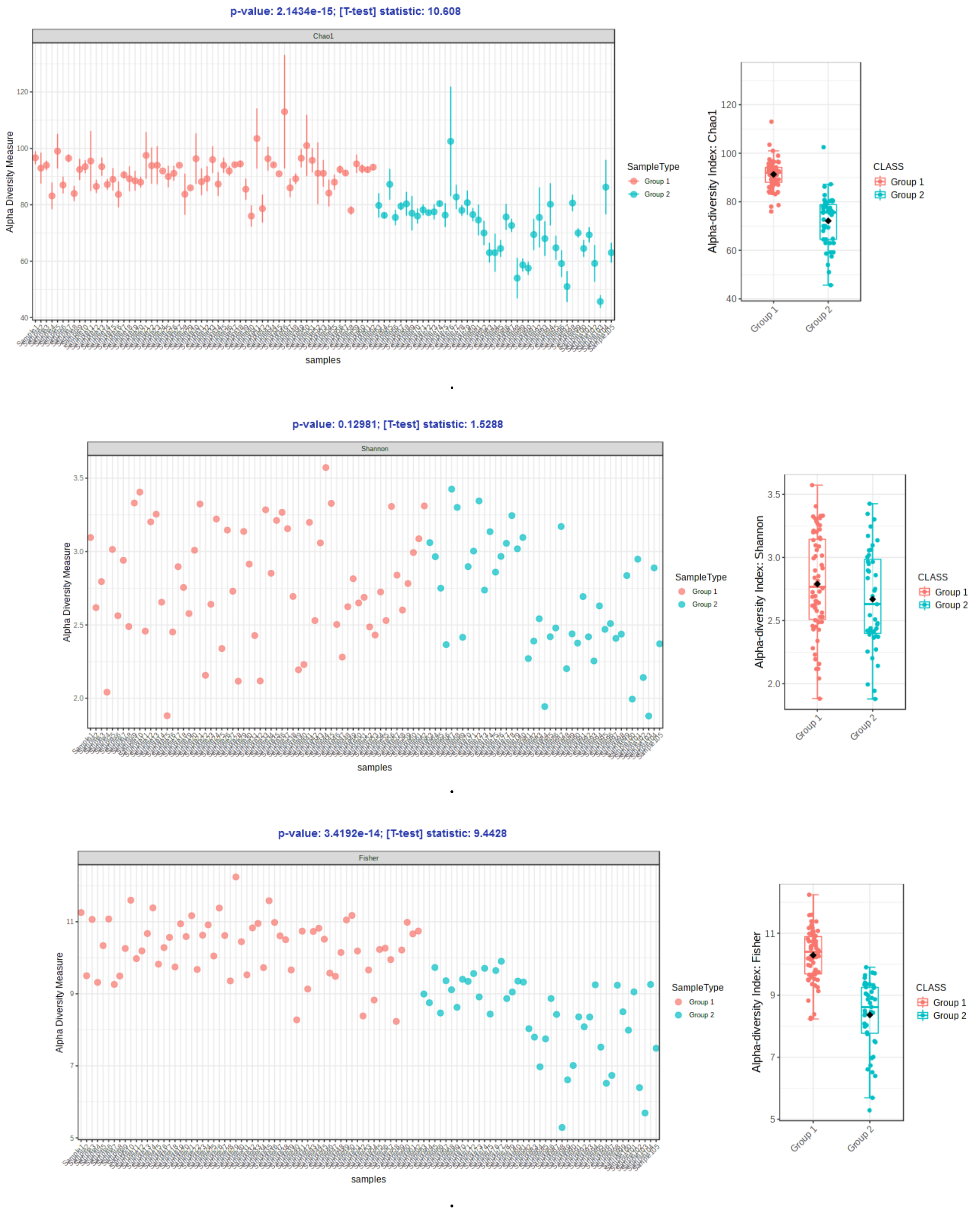

Figure S2. Alpha diversity for fecal microbiota.

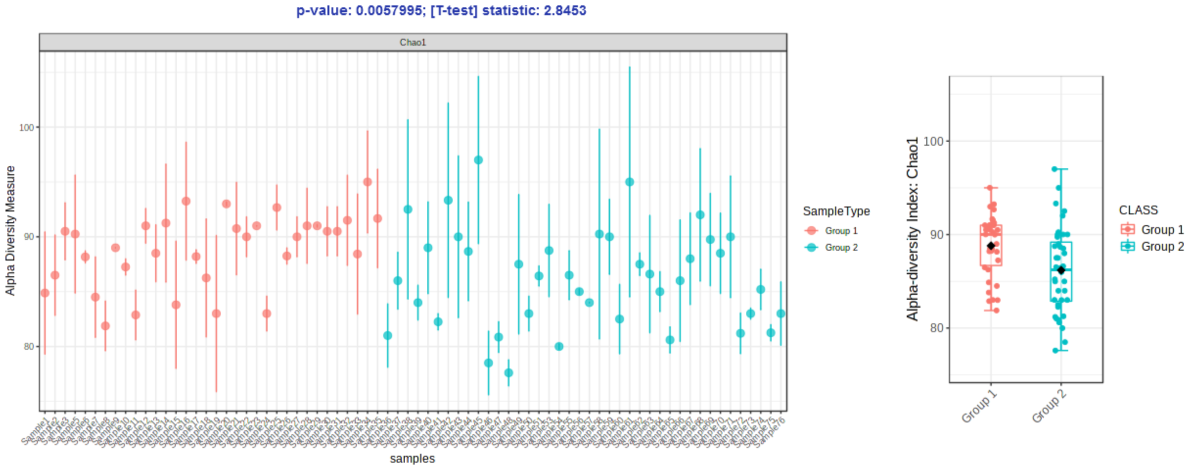

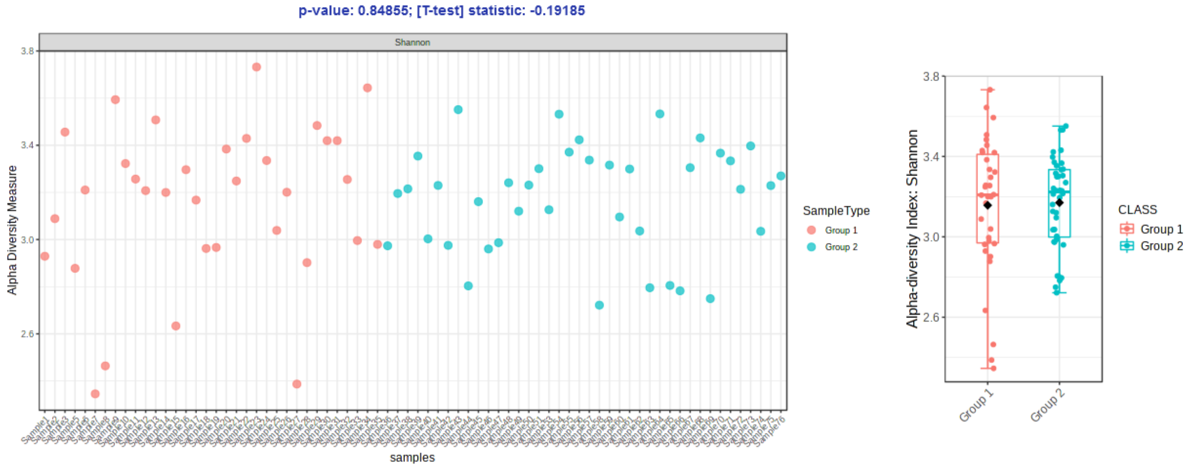

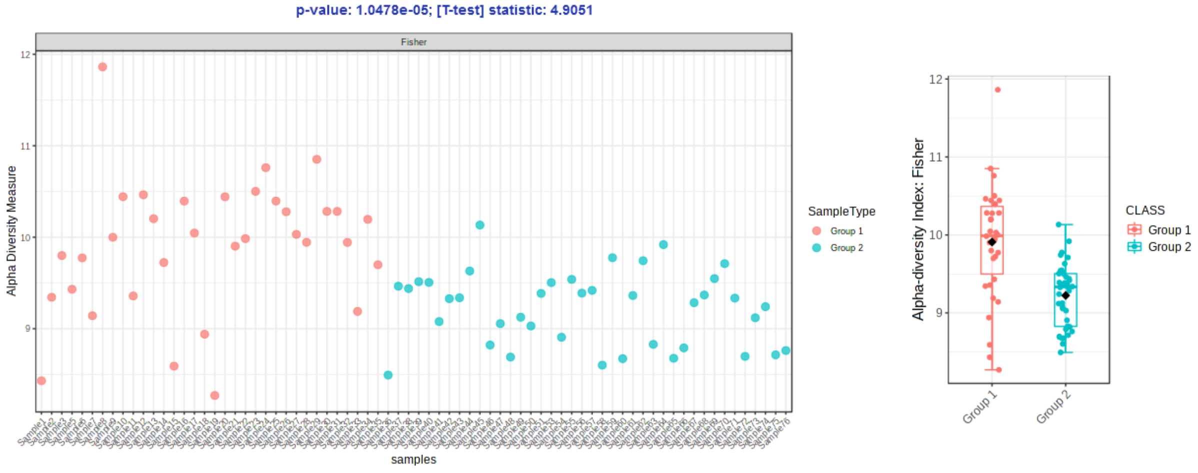

Figure S3. Beta diversity for oral microbiota.

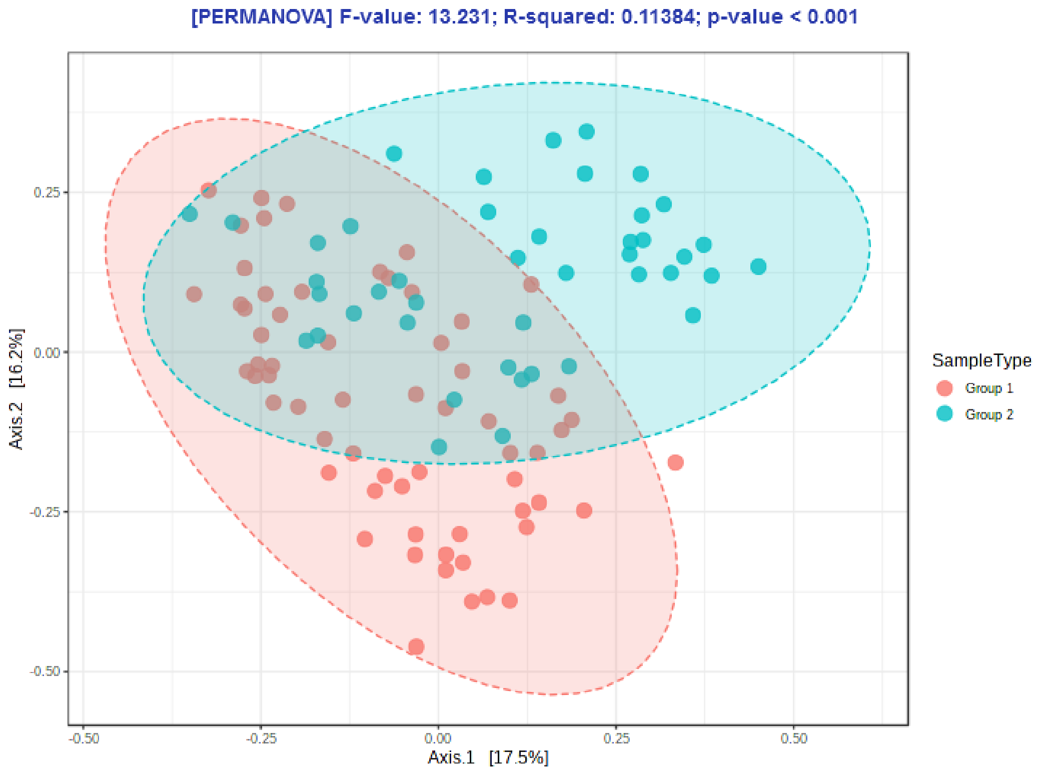

.

Figure S4. Beta diversity for fecal microbiota.

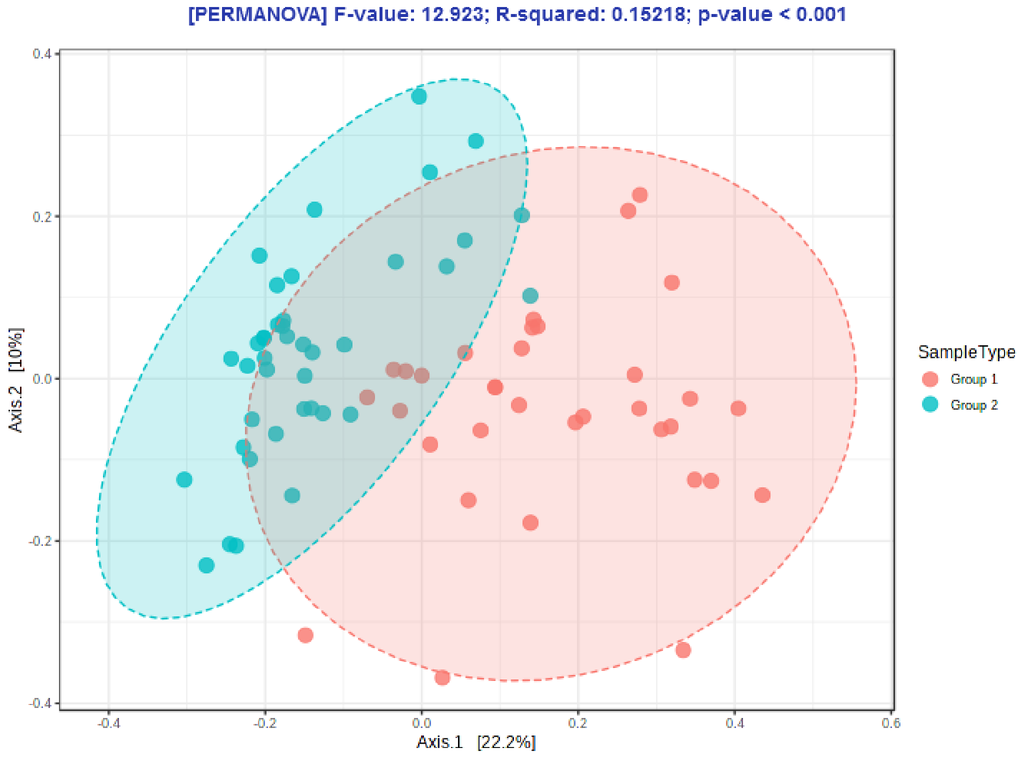

Supplement: Supplementary file 1 [file medicina-57-00678-s001.zip › medicina-1248326-supplementary.pdf]
